# Relationship of Salivary Microbiome with the Worsening of the Periodontal Health Status in Young Adults: A 3-Year Cohort Study

**DOI:** 10.3390/ijerph17051764

**Published:** 2020-03-09

**Authors:** Md Monirul Islam, Daisuke Ekuni, Naoki Toyama, Terumasa Kobayashi, Kohei Fujimori, Yoko Uchida, Daiki Fukuhara, Ayano Taniguchi-Tabata, Kota Kataoka, Yoshiaki Iwasaki, Manabu Morita

**Affiliations:** 1 Department of Preventive Dentistry, Okayama University Graduate School of Medicine, Dentistry and Pharmaceutical Sciences, Okayama 700-8558, Japan; p3a99o50@s.okayama-u.ac.jp (M.M.I.); pu171qxi@s.okayama-u.ac.jp (N.T.); de421015@s.okayama-u.ac.jp (T.K.); kfujimori@s.okayama-u.ac.jp (K.F.); de18017@s.okayama-u.ac.jp (K.K.); mmorita@md.okayama-u.ac.jp (M.M.); 2 Department of Preventive Dentistry, Okayama University Hospital, Okayama 700-8558, Japan; de20006@s.okayama-u.ac.jp (Y.U.); de20041@s.okayama-u.ac.jp (D.F.); de19026@s.okayama-u.ac.jp (A.T.-T.); 3 Health Service Center, Okayama University, Okayama 700-8530, Japan; yiwasaki@okayama-u.ac.jp

**Keywords:** salivary microbiome, periodontal health status, oral hygiene, cohort study, young adults

## Abstract

The purpose of this prospective cohort study was to investigate the influence of the salivary microbiome on the worsening of the periodontal health status among Japanese young adults. We assessed the data of systemically healthy and non-smoking young (18–22 years) university students (*n* = 457) from Okayama University at baseline (2013) and follow-up (2016). The worsening group was defined based on an increase in the percentage of bleeding on probing (%BOP) or an increase in probing pocket depth (PPD) from <4 mm to ≥4 mm. Unstimulated saliva samples were randomly collected from 69 students for microbiome analysis at follow-up. The salivary microbiome was assessed through 16S rRNA metagenomic sequencing. The type of community in the salivary microbiome clustered by statistical analysis and diversity was not significantly associated with the worsening of the periodontal health status in cases of increasing %BOP and PPD (*p* > 0.05). The prevalence of some species was significantly higher in the worsening group than in the non-worsening group (*p* < 0.05) in both cases. The worsening of the periodontal health status was associated with some species, but not the type of community and diversity in the salivary microbiome among Japanese young adults.

## 1. Introduction

The human oral cavity is colonized by numerous microbiomes [1]. Several studies have indicated that oral microbiome is related to oral infectious diseases, including caries, periodontal diseases, endodontic infections, alveolar osteitis, and tonsillitis [2,3].

The contribution of oral microbiome to periodontal diseases (i.e., gingivitis and periodontitis) is well known, and some pathogens have been identified among certain bacterial species or groups of species in the subgingival microbiome. Among them, three bacterial species, *Porphyromonas gingivalis*, *Tannerella forsythia,* and *Treponema denticola* (known collectively as the ‘red complex’), are strongly associated with periodontitis [4]. Besides, a systematic review of open-ended 16S rRNA gene analyses suggested a positive association of at least 17 novel species or phylotypes with periodontitis [5]. However, susceptibility to periodontitis strongly differs between individuals who harbor the same pathogenic bacterial profile [6]. Moreover, the roles of these species in the onset and the worsening of the periodontal health status are still unclear.

Periodontal pathogens detached from the subgingival microbiome are also identified in saliva [7,8,9,10,11]. Their presence or absence was reported to be associated with periodontal status [12]. Furthermore, the salivary microbiome detected by 16S rRNA gene analyses has the potential to reflect subgingival plaque-derived bacteria representing periodontal diseases [13]. Besides, 16S r RNA gene analysis has been used extensively for classification and identification of the salivary microbiome. However, the relationship between the salivary microbiome and the worsening of the periodontal health status detected by 16S rRNA gene analyses is not well characterized.

Bleeding on probing is an earlier and more sensitive indicator of inflammation than visual signs of inflammation (redness and swelling) [14]. It represents the most useful clinical predictor for the worsening of the periodontal health status [15]. On the other hand, measuring probing pocket depth (PPD) is reliable for assessing the worsening of periodontal health status among young adults [15]. Furthermore, more than 82% of American adolescents and more than 30% of Japanese young adults are affected by gingivitis [16,17]. In particular, 20.9–22.7% of Japanese university students had a PPD ≥4 mm [15]. Hence, we focused on the worsening of the periodontal health status among young adults. We hypothesized that the salivary microbiome profile is associated with the worsening of the periodontal health status. Therefore, the objective of our study was to determine whether the salivary microbiome is associated with the worsening of the periodontal health status among Japanese young adults.

## 2. Materials and Methods

### 2.1. Ethics Statement

The study was approved by the Ethics Committee of Okayama University Graduate School of Medicine, Dentistry and Pharmaceutical Sciences (No. 1060). All methods were performed in accordance with the Helsinki declaration. All participants understood the nature of the study and provided informed consent.

### 2.2. Study Participants

**Figure 1:**
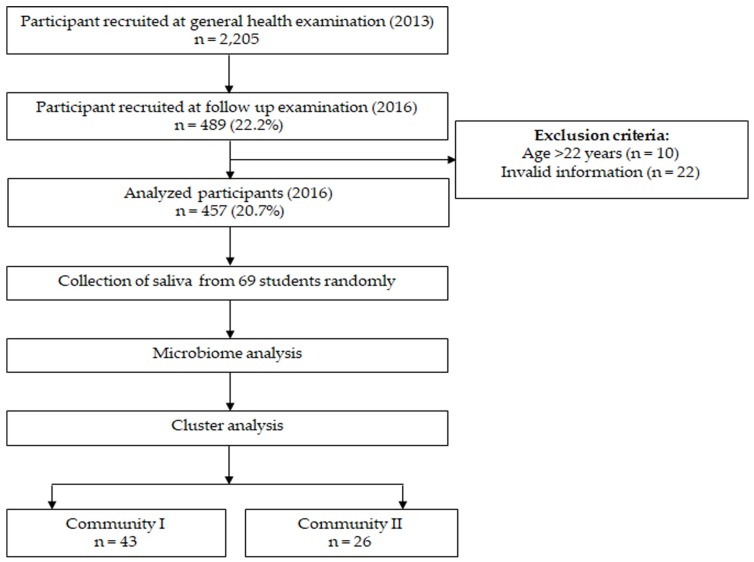
Flowchart of the study. Two communities were identified using k means clustering based on the salivary microbiome.

The inclusion criteria were young adults (18–22 years old) who were systemically healthy and non-smokers, completed the questionnaire, received both the baseline evaluation in April 2013 and 3-year follow-up examination before graduation in April 2016 at the Health Service Center in Okayama University and agreed to participate in the study. We excluded participants who provided incomplete responses in the questionnaire (Figure 1).

### 2.3. Oral Examination

The oral health condition of all participants who participated in this study was examined by five calibrated dentists (Daisuke Ekuni, Ayano Taniguchi-Tabata, Shinsuke Mizutani, Mayu Yamane-Takeuchi, and Kota Kataoka). For each participant, the periodontal condition was assessed using the Community Periodontal Index (CPI) (World Health organization, 4th edition) by a CPI probe (YDM, Tokyo, Japan) at six sites on each tooth (mesio-buccal, mid-buccal, disto-buccal, disto-lingual, mid-lingual, and mesio-lingual) [18]. Ten teeth were selected for periodontal examination: two molars in each posterior sextant and the upper right and lower left central incisors. The percentage of teeth exhibiting bleeding on probing (%BOP) among the ten examined teeth was calculated [17]. The simplified oral hygiene index (OHI-S) was used to assess the level of dental plaque and calculus [19]. The buccal surfaces and the lingual surfaces of the index teeth was examined to obtain the OHI-S index scores. By adding the recorded scores and dividing it by the number of surfaces examined the debris and calculus scores were calculated, respectively. Finally, the OHI-S score was obtained by adding the Debris Index (DI-S) and Calculus Index (CI-S) score. The decayed, missing, and filled teeth (DMFT) score was recorded (World Health organization, 4th edition). After training the examiners, the PPD in the 10 teeth used for CPI was recorded and repeated within a 2-week interval in two volunteers. The intra- and inter-examiner reliability was determined by the κ statistic (>0.8).

### 2.4. Assessment of Body Mass Index (BMI)

The previous study among young adults mentioned that BMI can affect their periodontal condition [15]. Therefore, in the general health examination, the height and body weight of participants were measured by the university’s public health nurses using the Tanita body fat analyzer (Model No. BF-220; Tanita, Tokyo, Japan). BMI was computed as weight in kilograms divided by height in meters squared [20].

### 2.5. Health-Related Questionnaire

Participants were asked about their general health condition and lifestyle habits during baseline and follow-up examinations using a questionnaire, which included age, sex, the history of any disease, medication, and smoking status. Additionally, participants were asked about the following oral health behaviors: teeth brushing frequency (≥2 times/<2 times), dental floss use (yes/no), and regular dental checkup over the last year (yes/no) [21,22] because these are related with periodontal health.

### 2.6. Salivary Microbiome Analysis

Unstimulated saliva samples were randomly (a random number list) collected from 69 students for microbiome analysis at follow up (2016). Before the dental examination, saliva (1 mL) samples were collected from the participants in a sterile plastic tube (from 09:00 to 16:00). Samples were stored at −80 °C until further analysis.

DNA was extracted from each collected sample using the QIAamp DNA Mini kit (QIAGEN, Hilden, Germany) according to the manufacturer’s instructions. Subsequently, the V3 and V4 regions of the 16S rRNA gene were amplified using primers 357F (5′-TCGTCGGCAGCGTCAGATGTGTATAAGAGACAGCCTACGGGNGGCWGCAG-3′) and 781R (5′-GTCTCGTGGGCTCGGAGATGTGTATAAGAGACAGGACTACHVGGGTATCTAATCC-3′). The purified amplicons were sequenced on the MiSeq platform (MiSeq Reagent V3 600 cycles, Illumina, San Diego, CA, USA) at Okayama University Hospital Biobank (Okayama University Hospital, Okayama, Japan) according to the standard protocol.

Thereafter, obtained raw sequences data were screened, trimmed, filtered, denoised, and barcoded. Sequenced data were analyzed with USEARCH (version 8.0.1623, https://www.drive5.com/usearch/) at the Oral Microbiome Center (Taniguchi dental clinic, Kagawa, Japan). Next, the chimeric sequences were depleted from the dataset using the UCHIME algorithm, which imparts faster, highly sensitive, and accurate chimera detection [23]. We discarded sequences for analysis if they were shorter than 400 bases [24]. At a 97% level of nucleotide similarity cut-off, the operational taxonomic units (OTUs) were sorted applying the UCLUST algorithm. Next, obtained sequences were analyzed to identify oral taxa against the Human Oral Microbiome Database (HOMD; version 14.5) (http://www.homd.org/) [25].

### 2.7. Statistical Analysis

The sample size for the saliva examination was estimated from a previous study [26]. Wherein, %BOP was selected as the primary outcome. Based on the data, the minimum sample size required was 54 to provide a power of 95% with an alpha of 0.05 by *t*-test. To calculate the effect size, mean difference (4.6), and average SD (19.9) for the BOP of the two communities were considered. In our study, we used G*Power (version 3.1.9.2, Düsseldorf, Germany) statistical power analysis tool to determine sample size [27]. The normality of the data was confirmed by a quantile–quantile plot. Comparisons of demographics and clinical parameters between baseline and follow-up and between the worsening and non-worsening groups were performed by the paired *t*-test, unpaired *t*-test, chi-square test, or Mann–Whitney *U* test as applicable. *p* < 0.05 was considered statistically significant. The worsening of periodontal health status was defined based on the increase in %BOP and increase in PPD from <4 mm to ≥4 mm. To assess the inter-individual variation of salivary microbiome among our subjects besides the worsening and non-worsening group, two communities (among the saliva examined group; *n* = 69) were constructed based on the k means clustering algorithm; an unsupervised learning algorithm. Two groups were categorized based on the mean percentage of the salivary microbiome. Principal component analysis was used to analyze the clustering of participants. The relative abundance of 28 OTUs with mean relative abundances ≥1% were utilized to create the co-occurrence networks following Spearman’s rank-correlation coefficients test. Then, co-occurrence networks were generated using the ‘igraph’ package in R (version 3.4.3; The R Project for Statistical Computing, http://www.R-project.org). Alfa diversity indices of all species were calculated among the worsening, non-worsening groups, and the two communities. Finally, we calculated the odds ratio (OR) and 95% confidence interval (CI) using a logistic regression model, where an increase in %BOP and PPD were considered as dependent variables. Sex, oral health behaviors at baseline, BMI, community type, and OHI-S score were included as independent variables. All statistical analyses were performed using STATA (version 13.1; StataCorp, College Station, TX, USA).

## 3. Results

### 3.1. Participants’ Characteristics

Figure 1 shows the study flowchart. Of 2205 first-year students who volunteered to receive an oral examination and completed the questionnaire at the Health Service Center, Okayama University, in April 2013, 489 students received a 3-year follow-up examination before graduation in April 2016 (follow-up rate = 22.2%). Data from 32 students were excluded due to invalid information and age >22 years. Therefore, 457 healthy participants (non-antibiotic takers) (217 males and 240 females) with a mean age of 18.2 ± 0.43 years at baseline were finally analyzed in this study.

There was a significant difference (*p* < 0.05) in dental floss use, %BOP, BMI, and PPD ≥4 mm between baseline and follow-up of these participants (Table 1).

When we defined the worsening of the periodontal health status as an increase in %BOP, a significant difference was found in OHI-S scores at baseline between the worsening and non-worsening groups (Table S1). Additionally, among participants (*n* = 69) who provided salivary samples, OHI-S scores were also significantly different between the worsening and non-worsening groups (*p* < 0.05) (Table 2).

When we defined the worsening of the periodontal health status as PPD ≥4 mm, there were significant differences in OHI-S score, %BOP, daily brushing frequency, and PPD at baseline between the worsening and non-worsening groups (*p* < 0.05) (Table S2). No significant differences were found in variables among participants who provided salivary samples (*n* = 69) (Table 2). Furthermore, logistic regression analysis revealed that the risk of increased %BOP after 3 years was significantly influenced by sex (*p* = 0.028) and OHI-S score (*p* < 0.001). By contrast, the risk of increased PPD after 3 years was significantly influenced by PPD ≥4 mm at baseline (*p* < 0.006) (Table S3).

### 3.2. Comparison of the Salivary Microbiome Profiles

Using 16S rRNA metagenomic sequencing analysis, we obtained a total of 10,915,034 reads. Among them, 4,155,030 quality-passed reads (mean ± SD: 60,217 ± 17,205) from regions V3 and V4 of the bacterial 16S rRNA gene were used for analysis. The sequences were assigned to 203 species-level OTUs. There were 73 genera and 13 phyla. Of them, Firmicutes (30%), Bacteroidetes (21%), Proteobacteria (14%), Actinobacteria (9%), Fusobacteria (6%), TM7 (6%), Spirochaetes (3%), Low identity (3%), GNO2 (3%), Chloroflexi (2%), SR1 (1%), Synergistetes (1%). and Tenericutes (1%) were major components (≥1%). *Streptococcus sp*. and *TM7 [G-1]* were most abundant in all participants.

Subsequently, species of the salivary microbiome in both groups (worsening and non-worsening) associated with increased %BOP and PPD were compared. Notably, the prevalences of *Campylobacter rectus*, *Dialister invisus*, *Prevotella shahii*, *Streptococcus parasanguinis*, and *Dialister pneumosintes* were significantly higher in the worsening group than in the non-worsening group (*p* < 0.05) in cases of increasing %BOP. The prevalences of *Streptococcus salivarius*, *Prevotella histicola*, *Selenomonas* sp., *Lachnoanaerobaculum orale*, *Stomatobaculum longum*, *Prevotella nigrescens*, *Actinomyces lingnae*, *Actinomyces oris*, and *Actinobaculum* sp. were significantly higher in the worsening group than those in the non-worsening group (*p* < 0.05) in cases of increasing PPD.

There were no significant differences in the presence/prevalence of the red complex (*Porphyromonas gingivalis*, *Tannerella forsythia*, and *Treponema denticola*) between the worsening and non-worsening groups (*p* > 0.05), although more than 50% of participants had these species (data not shown).

**Figure 2:**
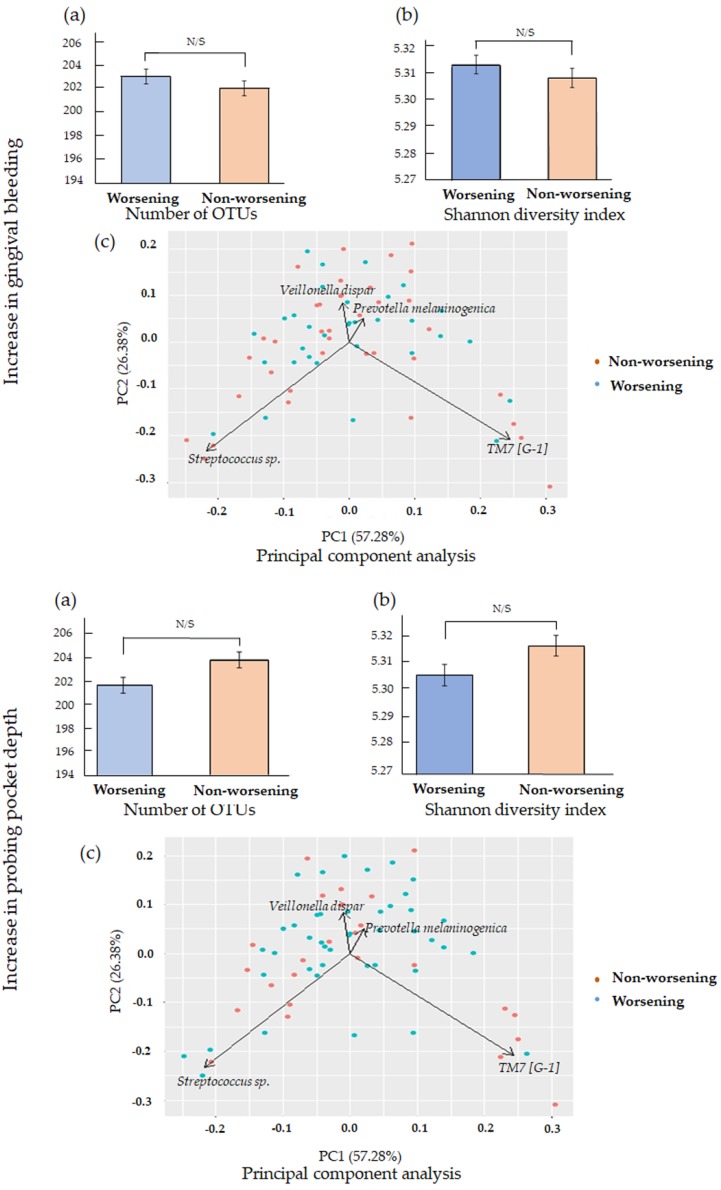
(**a**) Mean number of operational taxonomic units (OTUs); (**b**) Shannon diversity index values and (**c**) Principal component analysis (PCA) of 69 participants (increase in gingival bleeding and increase in probing pocket depth (PPD)). There were no significant differences in the number of OTUs, Shannon diversity index values, and PCA between the worsening and non-worsening groups.

We further investigated whether any significant difference in the number of OTUs and Shannon diversity index values by comparing the worsening and non-worsening groups. Besides, the PCA was also conveyed to demonstrate the similarity of microbiome composition for both groups. However, the difference did not reach significance, and the PCA shows the similarity of microbiomes’ distribution (Figure 2).

### 3.3. Microbiome Community Characterization

We classified all participants into two communities (community I and II) through k means cluster analysis based on the relative abundance of all species. Participants’ general and clinical parameters were not significantly different between the two communities (Table S4). Additionally, there were no significant differences in the number of OTUs and Shannon diversity index values between the two communities (Figure S1).

PCA was employed to assess the distribution of both communities based on all species. Community I and community II appeared to cluster together in opposing directions. Furthermore, *Streptococcus* sp. and *Prevotella melaninogenica* appeared to be associated with community I, while *TM7 [G-1]* and *Veillonella dispar* appeared to be associated with community II (Figure S1).

Moreover, we determined 28 predominant species (mean ≥1%) that estimated 72.1% of the total microbiome to construct a microbial co-occurrence network based on Spearman’s correlation coefficient. There were two cohabiting groups among our participants. The species in cohabiting group I included *TM7 [G-3]*, *TM7 [G-1]*, *Prevotella pallens*, *Prevotella melaninogenica*, *Prevotella histocola*, *Butyrivibrio* sp., *Prevotella salivae*, *Prevotella* sp., *Selemonas* sp., *Velillonella atypica*, *Megasphaera micronuciformis*, *Veillonella dispar*, *Oribacterium sinus*, *Ruminococcaceae [G-2],* and *Ruminococcaceae [G-1]*), and in cohabiting group II included *Campylobactor showae*, *Campylobactor rectus*, *Fusobacterium periodonticum*, *Leptotrichia* sp., *Porphyromonas* sp., and *SR1 [G-1*]) (Figure S2).

## 4. Discussion

Several cross-sectional studies showed that a relationship exists between oral microbiome and periodontal diseases among various age groups [28,29,30,31]. However, few cohort studies have investigated the relationship between them.

The type of community in the salivary microbiome clustered by statistical analysis was not significantly associated with the worsening of the periodontal health status in this study. Community I included a higher percentage of *Streptococcus* sp., while community II primarily included *TM7 [G-1]*. Previous cross-sectional studies showed that the abundance of *TM7* was higher in sites with mild periodontitis than in healthy sites [32,33]. However, this study showed that community II was not significantly associated with the worsening of the periodontal health status (for an increase in %BOP and PPD), although a higher proportion of *TM7* was observed with increase in PPD (worsening group: 69.8% vs. non-worsening group: 50.0%). Furthermore, our finding was inconsistent with a previous cross-sectional study that showed a significant association between salivary microbiome communities and periodontal diseases status [26]. This discrepancy may be explained by differences in study design (prospective cohort study vs. cross-sectional study) and targeted age group (young adults vs. elderly people) [34,35]. The previous studies mentioned that the composition of salivary microbial profiles could be different in relation to age [26,36]. Further cohort studies are required to investigate the relationship between a specific community in the salivary microbiome and the worsening of the periodontal health status.

The diversity in the microbiome in saliva was also not associated with the worsening of the periodontal health status in this study. On the contrary, a recent study suggests that salivary microbiome diversity is an important aspect of periodontal diseases, and there is an estimated association between the loss of diversity and the progression of periodontal diseases [37]. Another study mentioned the association between subgingival microbiome diversity and periodontal disease [33]. Therefore, when collecting subgingival plaque instead of saliva, the diversity might be observed between the worsening and non-worsening groups.

We also evaluated the difference in the prevalence of each bacterium between the worsening and non-worsening groups. Among them, the prevalences of *Streptococcus salivarius*, *Prevotella histicola*, *Selenomonas* sp., *Lachnoanaerobaculum orale*, *Stomatobaculum longum*, *Prevotella nigrescens*, *Actinomyces lingnae*, *Actinomyces oris*, and *Actinobaculum* sp. were significantly higher in the worsening group than in the non-worsening group. Our results partially support those of a previous study [35], which found that *Prevotella* was associated with periodontal diseases. Other species in our study may indirectly contribute to the worsening of the periodontal health status. However, an association between these species and periodontal disease was not observed in other studies [5,38].

Periodontal pathogenic bacteria, including the red complex, were not associated with the worsening of the periodontal health status among young adults in this study. However, these pathogens are shown to be strongly associated with periodontal diseases [4]. The discrepancy in our findings may be explained by the periodontal status of young adults (non-severe cases). Further cohort studies are required to investigate the relationship between the certain salivary microbiome and the worsening of the periodontal health status in young adults with severe periodontitis.

In this study, the OHI-S score at baseline was negatively associated with the worsening of the periodontal health status (increase in %BOP). A low OHI-S score at baseline contributes to the worsening of the periodontal health status. One possible reason may depend on the worsening change in oral hygiene in students with low OHI-S scores during the study period. As a result, students with low OHI-S scores showed worsening of the periodontal health status, as noted by the increase in OHI-S scores.

In Table 2, %BOP at baseline in the worsening group was significantly lower than that in the non-worsening group (14.4 ± 13.9 vs. 39.2 ± 24.2). During three years, the %BOP in the worsening group significantly increased. On the other hand, the %BOP in the non-worsening group significantly decreased. Thus, the %BOP in the worsening group was significantly higher than that in the non-worsening group at follow-up (43.44 ± 18.42 vs. 24.1 ± 19.1, *p* < 0.001), which can explain the discrepancy in %BOP at baseline between the two groups.

Our results confirmed that PPD at baseline was an important predictor of the worsening of the periodontal health status. A previous study suggested that the number of sites with PPD ≥5 mm was identified as a risk predictor regarding periodontal diseases progression [39,40]. Our results support this previous finding.

Our results showed that avoiding maintaining good oral health behaviors (using dental floss and regular dental checkup) worsened the %BOP of young adults. The previous studies already mentioned oral health behaviors as an important factor for controlling gingival bleeding among young adults [15,18]. Therefore, it is essential to encourage them to maintain good oral health behaviors, which contribute to controlling the gingival bleeding or further periodontal disease progression.

Smoking, diabetes, obesity, and other risk factors for periodontal diseases are also associated with components of the oral microbiome and dysbiosis [10,41,42,43,44]. However, none of the participants in this study were smokers, obese, or had systemic diseases. Furthermore, compositional transitions in the oral microbiome can be observed by external stresses throughout life [45]. As our study period was short (3 years), further long-term studies are needed to investigate the interaction between the salivary microbiome and host changes.

We used salivary samples to investigate the oral microbiome because of its advantages. Saliva can be easily, repetitively, and noninvasively collected. Saliva may be a reasonable surrogate for pooled subgingival samples when screening for the presence of periopathogens and reflect periodontal conditions [35,46]. Further research using both salivary and dental plaque samples is needed for better understanding of the influence of the oral microbiome on the worsening of the periodontal health status among young adults.

Our study has some limitations. First, the follow-up rate was low (20.7%) because only a few students received 3-year follow-up examinations before graduation. To check for sampling bias, we examined the general and oral health conditions of all subjects at baseline and looked for differences between subjects who received the 3-year follow-up examinations and those who did not. No significant differences were observed. Thus, the sampling bias might be small in this study. Second, all participants were recruited from Okayama University. This may limit the generalizability of our findings. Third, we only used salivary samples to investigate the oral microbiome but not plaque. Fourth, no specific indication was given to the participants prior to saliva collection. As we randomly collected the saliva, and participants did not eat any food and drink nor brush their teeth within the last 30 min, the effects of pattern and timing of collection on the results might be small. However, unknown factors that remained in the oral cavity might have affected the results. Finally, we did not examine the microbiome in saliva at baseline. Several studies show the stable condition of the oral microbiome except for the rare microbiome [47,48,49]. Since periodontal treatment can change the subgingival microbiome composition [50,51,52], we checked and confirmed that the results were similar even when participants who received a regular dental checkup for periodontal treatment were excluded. Although there might be the impact of a regular dental checkup for the subgingival microbiome composition, it may be small for the salivary microbiome among young adults in this study. Moreover, due to cost limitation, we only analyzed the salivary microbiome of only randomly selected participants at follow up. The results should be considered with caution.

## 5. Conclusions

In conclusion, our 3-year cohort study suggested that the worsening of the periodontal health status was associated with some species, but not the type of community and diversity in the salivary microbiome among Japanese young adults.

## Figures and Tables

**Table 1:** Demographics and change in parameters from baseline to follow-up among all students (*n* = 457).

Co-variate	Baseline	Follow-Up	*p*-Value
Age (years)	18.2 ± 0.4 ^1^	–	–
Sex (Male)	217 (47.5) ^2^	–	–
Daily brushing frequency (≥2 times)	374 (81.8)	387 (84.7)	0.249 ^3^
Daily flossing (Yes)	26 (5.7)	68 (15.1)	<0.001 ^3^
Regular dental checkup (Yes)	70 (15.3)	66 (14.4)	0.643 ^3^
OHI-S score	0.6 ± 0.6	0.6 ± 0.7	0.881 ^4^
BOP (%)	28.7 ± 24.2	31.4 ± 23.0	0.040 ^4^
DMFT	2.3 ± 2.8	2.9 ± 2.7	<0.001 ^4^
BMI (kg/m^2^)	20.7 ± 2.8	21.0 ± 2.9	<0.001 ^4^
Community periodontal index (CPI)			
0	50 (10.9)	26 (5.7)	<0.001 ^5^
1	115 (25.2)	88 (19.3)	
2	223 (48.8)	128 (28.0)	
3	69 (15.1)	199 (43.5)	
4	0 (0.0)	16 (3.5)	
PPD ≥4 mm (Yes)	69 (15.2)	215 (47)	<0.001 ^3^

OHI-S, simplified oral hygiene index; BOP, bleeding on probing; DMFT, decayed, missing, and filled teeth; BMI, body mass index; PPD, probing pocket depth. ^1^ mean ± standard deviation, ^2^ number (%), ^3^ McNemar test, ^4^ paired *t*-test, ^5^ chi-square test.

**Table 2:** Comparison between worsening and non-worsening groups (increase in bleeding on probing (BOP) and probing pocket depth) among randomly selected young adults (*n* = 69).

At Baseline	Increase in Gingival Bleeding			Increase in Probing Pocket Depth		
	Worsening (*n* = 32)	Non-Worsening (*n* = 37)	*p*-Value	Worsening (*n* = 43)	Non-Worsening (*n* = 26)	*p*-Value
Age (years)	18.2 ± 0.4 ^1^	18.2 ± 0.5	0.417 ^3^	18.2 ±0.5	18.2 ± 0.4	0.689 ^3^
Sex (Male)	7 (21.9) ^2^	14 (37.9)	0.151 ^4^	11 (25.6)	10 (38.5)	0.260 ^4^
OHI-S score	0.3 ± 0.4	0.7 ± 0.6	0.003 ^3^	0.6 ± 0.6	0.4 ± 0.3	0.116 ^3^
BMI (kg/m^2^)	19.9 ± 2.7	20.2 ± 2.9	0.731 ^3^	20.3 ± 2.9	19.7 ± 2.7	0.434 ^3^
BOP (%)	-	-	-	29.1 ± 23.2	25.4 ± 24.2	0.532 ^3^
DMFT (number)	1.6 ± 2.3	2.7 ± 3.3	0.101 ^3^	2.6 ± 3.2	1.5 ± 2.3	0.103 ^3^
Daily brushing frequency (≥2 times)	29 (90.6)	30 (81.1)	0.261 ^4^	36 (83.7)	23 (88.5)	0.588 ^4^
Daily flossing (Yes)	4 (12.5)	2 (5.4)	0.296 ^4^	5 (11.6)	1 (3.8)	0.266 ^4^
Regular dental checkup (Yes)	8 (25.0)	9 (24.3)	0.948 ^4^	12 (27.9)	5 (19.2)	0.418 ^4^
CPI						
0	4 (12.5)	2 (5.4)	0.348 ^4^	2 (4.7)	4 (15.4)	0.186 ^4^
1	7 (21.9)	9 (24.3)		10 (23.3)	6 (23.1)	
2	11 (34.4)	19 (51.4)		16 (37.2)	14 (53.8)	
3	10 (31.3)	7 (18.9		15 (34.9)	2 (7.7)	
4	0 (0.0)	0 (0.0)		0 (0.0)	0 (0.0)	
PPD ≥4 mm (Yes)	10 (31.3)	7 (18.9)	0.236 ^4^	-	-	-
Daily brushing frequency (Increased)	8 (25.0)	7 (18.9)	0.541 ^4^	11 (25.6)	4 (15.4)	0.319 ^4^
Flossing (Increased)	1 (3.1)	10 (27.0)	0.006 ^4^	6 (13.9)	5 (19.2)	0.561 ^4^
Regular dental checkup (Increased)	0 (0.0)	6 (16.2)	0.016 ^4^	4 (9.3)	2 (7.7)	0.818 ^4^
Community I	20 (46.5)	23 (53.5)	0.906 ^4^	30 (69.8)	13 (30.2)	0.080 ^4^

OHI-S, simplified oral hygiene index; BMI, body mass index; BOP, bleeding on probing; DMFT, decayed, missing, and filled teeth; PPD, probing pocket depth. ^1^ mean ± standard deviation, ^2^ number (%), ^3^ unpaired *t*-test, ^4^ chi-square test.

## Supplementary Materials

The following are available online at https://www.mdpi.com/1660-4601/17/5/1764/s1, Table S1. Comparison between the worsening and non-worsening groups (increase in gingival bleeding) among all students (*n* = 457); Table S2. Comparison between the worsening and non-worsening groups (increase in probing pocket depth) among all students (*n* = 457); Table S3. Conditional odds ratios (ORs) and 95% confidence intervals (CIs) for an increase in gingival bleeding and probing pocket depth among all students (*n* = 457); Table S4. Comparisons between the two communities; Figure S1. Differences in the salivary microbiome between the two communities; Figure S2. Microbial co-occurrence networks of species.
