# Supplementary material for: Relationship of Salivary Microbiome with the Worsening of the Periodontal Health Status in Young Adults: A 3-Year Cohort Study
Source: Int J Environ Res Public Health. 2020 Mar 9;17(5):1764. doi: 10.3390/ijerph17051764 (PMC7085813; doi:10.3390/ijerph17051764)
Supplement: Supplementary file 1 [file ijerph-17-01764-s001.pdf]

Supplementary material

Table S1. Comparison between worsening and non- worsening groups (increase in gingival bleeding) among all students (n = 457).

At Baseline	Worsening (n = 211)	Non-Worsening (n = 246)	p -Value
Age (years)	18.2 ± 0.4 [1]	18.2 ± 0.4	0.778 [3]
Sex (Male)	108 (51.2) [2]	109 (44.3)	0.142 [4]
OHI-S score	0.4 ± 0.4	0.7 ± 0.6	<0.001 [3]
BMI (kg/m^2)	20.9 ± 3.1	20.5 ± 2.6	0.218 [3]
BOP (%)	18.3 ± 18.7	37.6 ± 24.7	<0.001 [3]
DMFT (number)	2.4 ± 2.9	2.2 ± 2.8	0.497 [3]
Daily brushing frequency (≥2 times)	169 (80.1)	205 (83.3)	0.371 [4]
Daily flossing (Yes)	10 (4.8)	16 (6.5)	0.417 [4]
Regular dental checkup (Yes)	32 (15.2)	38 (15.4)	0.934 [4]
PPD ≥4 mm (Yes)	29 (13.7)	40 (16.3)	0.454 [4]
Daily brushing frequency (Increased)	39 (18.5)	0 (16.3)	<0.001 [4]
Flossing (Increased)	16 (7.6)	37 (15.1)	0.013 [4]
Regular dental checkup (Increased)	11 (5.2)	31 (12.6)	0.006 [4]

OHI-S, simplified oral hygiene index; BMI, body mass index; BOP, bleeding on probing; DMFT, decayed, missing, and filled teeth; PPD, probing pocket depth. [1] mean ± standard deviation, [2] number (%), [3] unpaired *t*-test, [4] chi-square test.

Table S2. Comparison between worsening and non- worsening groups (increase in probing pocket depth) among all students (n = 457).

At Baseline	Worsening (n = 216)	Non-Worsening (n = 241)	p -Value
Age (years)	18.2 ± 0.4 [1]	18.2 ± 0.5	0.568 [3]
Sex (Male)	107 (49.5) [2]	110 (45.6)	0.405 [4]
OHI-S score	0.6 ± 0.5	0.5 ± 0.6	0.019 [3]
BMI (kg/m^2)	20.8 ± 3.1	20.6 ± 2.6	0.375 [3]
BOP (%)	31.3 ± 23.7	26.4 ± 24.3	0.030 [3]
DMFT (number)	2.6 ± 2.9	2.1 ± 2.7	0.221 [3]
Daily brushing frequency (≥2 times)	167 (77.3)	208 (86.3)	0.012 [4]
Daily flossing (Yes)	13 (6.0)	13 (5.4)	0.774 [4]
Regular dental checkup (Yes)	26 (12.0)	44 (18.3)	0.065 [4]
PPD ≥4 mm (Yes)	45 (20.8)	24 (9.9)	0.001 [4]
Changes in oral health behaviors after 3 years			
Daily brushing frequency (Increased)	23 (10.6)	27 (11.2)	0.849 [4]
Flossing (Increased)	19 (8.8)	10 (4.14)	0.041 [4]
Regular dental checkup (Increased)	17 (7.9)	25 (10.4)	0.355 [4]

OHI-S, simplified oral hygiene index; BMI, body mass index; BOP, bleeding on probing; DMFT, decayed, missing, and filled teeth; PPD, probing pocket depth. [1] mean ± standard deviation, [2] number (%), [3] unpaired *t*-test, [4] chi-square test.

Table S3. Conditional odds ratios (ORs) and 95% confidence intervals (CIs) for increase in gingival bleeding or probing pocket depth among all students (n = 457).

Dependent Variable	Independent Variable at Baseline		OR (95% CI) [1]	*p*-Value
Increase in BOP	Sex	Male	1	
		Female	0.65 (0.45–0.95)	0.028
	OHI-S score		0.32 (0.21–0.48)	<0.001
	BMI		1.03 (0.97–1.09)	0.301
	Daily brushing frequency	≥2 times	1	
		<2 times	0.85 (0.61–1.18)	0.338
	Daily flossing	Yes	1	
		No	1.46 (0.74–2.88)	0.275
	Regular dental checkup	Yes	1	
		No	1.05 (0.63–1.74)	0.849
	PPD ≥4 mm	No	1	
		Yes	0.88 (0.51–1.50)	0.632
Increase in PPD	Sex	Male	1	
		Female	0.92 (0.64–1.33)	0.685
	OHI-S score		1.33 (0.84–2.14)	0.221
	BMI		1.01 (0.96–1.08)	0.544
	Daily brushing frequency	≥2 times	1	
		<2 times	0.75 (0.54–1.04)	0.086
	Daily flossing	Yes	1	
		No	0.70 (0.36–1.37)	0.300
	Regular dental checkup	Yes	1	
		No	1.38 (0.83–2.31)	0.211
	PPD ≥4 mm	No	1	
		Yes	2.16 (1.24–3.75)	0.006

[1] Adjusted for sex, OHI-S, BMI, oral health behaviours and PPD ≥4 mm. Abbreviations: BOP, bleeding on probing; OHI-S, simplified oral hygiene index; BMI, body mass index; PPD, probing pocket depth.

Table S4. Comparisons between the two communities.

Parameters	Community I (n = 43)	Community II (n = 26)	*p*-Value
Age (years)	21.3 ± 0.5 [1]	21.1 ± 0.3	0.201 [3]
Sex (Male)	12 (27.9) [2]	9 (34.6)	0.557 [4]
Daily brushing frequency (≥2 times)	36 (83.7)	23 (88.5)	0.588 [4]
Daily flossing (Yes)	12 (27.9)	3 (11.5)	0.110 [4]
Regular dental checkup (Yes)	12 (27.9)	3 (11.5)	0.110 [4]
PPD ≥4 mm (Yes)	29 (67.4)	14 (53.8)	0.259 [4]
OHI-S score	0.4 ± 0.5	0.4 ± 0.6	0.943 [3]
BOP (%)	34.2 ± 20.6	31.2 ± 21.9	0.565 [3]
BMI (kg/m^2)	20.6 ± 3.3	20.4 ± 2.6	0.780 [3]

Abbreviations: OHI-S, simplified oral hygiene index; BOP, bleeding on probing; BMI, body mass index; PPD, probing pocket depth. [1] mean ± standard deviation, [2] number (%), [3] unpaired *t*-test, [4] chi-square test.

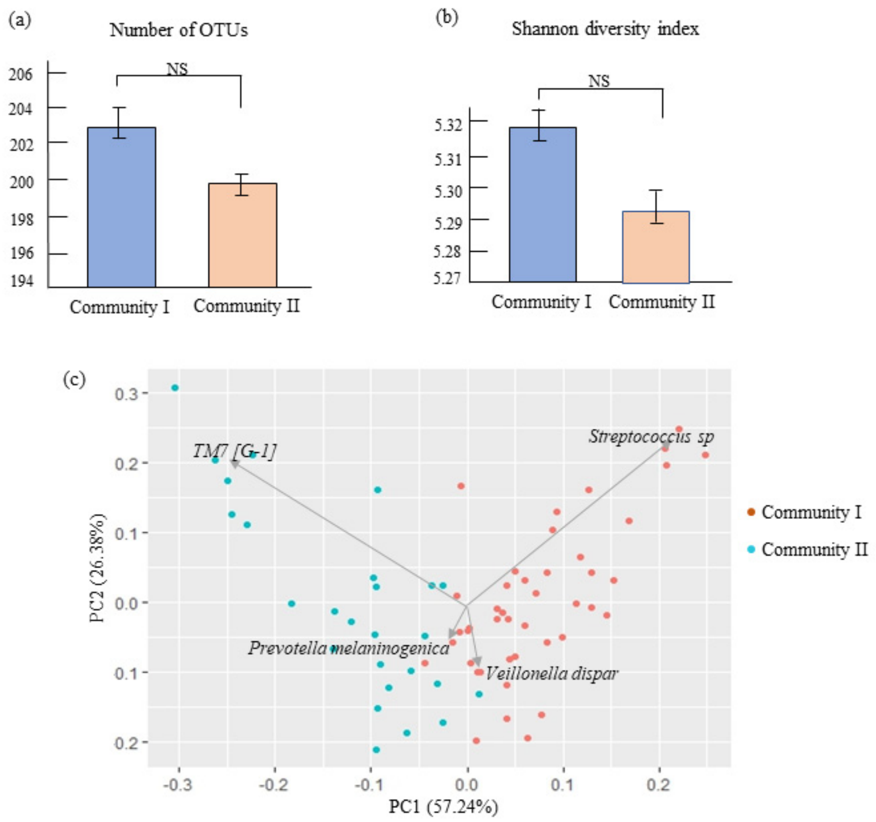

Figure S1. Differences in salivary microbiome between the two communities. (**a**) Mean number of operational taxonomic units (OTUs); (**b**) Shannon diversity index values and (**c**) Principal component analysis (PCA) of 69 participants (among two communities). There were no significant differences in number of OTUs, Shannon diversity index values and PCA between the community I and II.

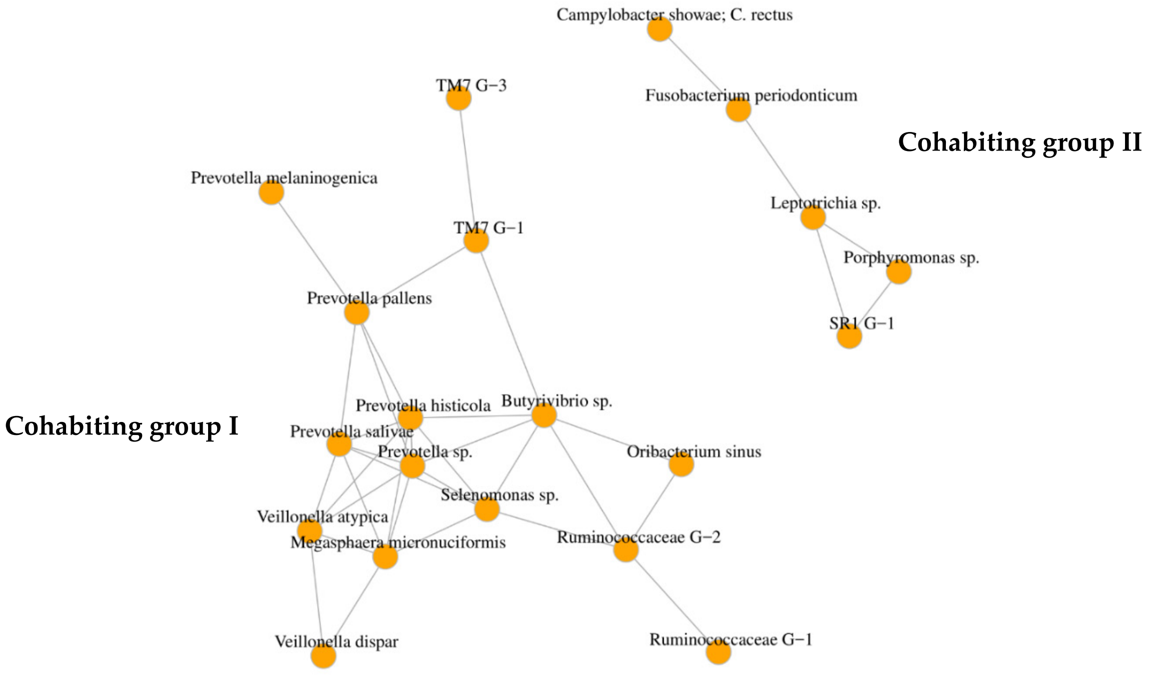

Figure S2. Microbial co-occurrence networks of species. Co-occurrence networks were constructed based on the relative abundances of 28 species (mean ≥1%). Each node represents species [in operational taxonomic units (OTUs)]. Only positive correlations between the two OTUs ($p< 0.001$ by Spearman's rank-correlation test) are displayed. (Cohabiting group I; *TM7 [G-3]*, *TM7 [G-1]*, *Prevotella pallens*, *Prevotella melaninogenica*, *Prevotella histocola*, *Butyrivibrio sp*., *Prevotella salivae*, *Prevotella sp*., *Selemonas sp*., *Velillonella atypica*, *Megasphaera micronuciformis*, *Veillonella dispar*, *Oribacterium sinus*, *Ruminococcaceae [G-2]* and *Ruminococcaceae [G-1].* Cohabiting group II; *Campylobactor showae*, *Campylobactor rectus*, *Fusobacterium periodonticum*, *Leptotrichia sp*., *Porphyromonas sp*. and *SR1 [G-1*]).
